# Kangxianhuanji formula and its component rutin ameliorate acute exacerbation of idiopathic pulmonary fibrosis by targeting GLUT1 to suppress HIF-1α–mediated glycolysis

**DOI:** 10.3389/fimmu.2026.1783256

**Published:** 2026-04-24

**Authors:** Siyuan Zhu, Wenjing Wu, Qin Zhang, Peng Zhao, Yan Du, Yunping Bai

**Affiliations:** 1 Lung Disease Diagnosis and Treatment Center, National Medical Center, The First Affiliated Hospital of Henan University of Chinese Medicine, Zhengzhou, China; 2 Collaborative Innovation Center for Chinese Medicine and Respiratory Diseases Co-constructed by Henan Province & Education Ministry of P.R. China/Henan Key Laboratory of Chinese Medicine for Respiratory Diseases, Henan University of Chinese Medicine, Zhengzhou, China

**Keywords:** acute exacerbation of idiopathic pulmonary fibrosis, glucose transporter 1, glycolysis, Kangxianhuanji formula, macrophage

## Abstract

**Background:**

Acute exacerbation of idiopathic pulmonary fibrosis (AE-IPF) is a life-threatening condition characterized by uncontrolled inflammation and progressive fibrosis, with limited effective therapies. Kangxianhuanji Formula (KHF), a traditional herbal prescription, has been used clinically for AE-IPF, but its molecular mechanisms remain unclear. This study aimed to elucidate the pharmacological mechanisms and active constituents of KHF.

**Methods:**

A bleomycin-induced AE-IPF mouse model was established to evaluate the therapeutic effects of KHF using histopathology, immunofluorescence, and inflammatory assessments. Network pharmacology was applied to predict targets, followed by drug affinity responsive target stability (DARTS) to identify direct binding proteins. Quantitative proteomics was used to validate target-related protein expression and pathway changes *in vivo*. Liquid chromatography–tandem mass spectrometry (LC–MS/MS) and molecular docking were used for compound–target analysis, and mechanistic validation was performed in macrophages using the glucose transporter 1 (GLUT1, encoded by SLC2A1) inhibitor STF-31 and cellular thermal shift assay (CETSA).

**Results:**

KHF markedly alleviated lung injury in AE-IPF mice, as shown by reduced collagen deposition, decreased levels of interleukin-1 beta (IL-1β), interleukin-6 (IL-6), tumor necrosis factor-alpha (TNF-α), and high mobility group box 1 (HMGB1), and suppression of abnormal proliferation of alveolar type II epithelial cells. Network pharmacology suggested involvement of glycolysis-related pathways, including PI3K–Akt and HIF-1α signaling. DARTS and proteomics consistently identified GLUT1 as a core target. KHF inhibited glycolytic reprogramming, reflected by reduced expression of GLUT1, HIF-1α, and hexokinase 2 (HK2), along with decreased lactate production. LC–MS/MS and molecular docking identified rutin as a key GLUT1-targeting compound, which was further confirmed by CETSA. In macrophages, rutin showed anti-inflammatory and anti-glycolytic effects, and co-treatment with STF-31 showed no additive effects, suggesting a GLUT1-dependent mechanism.

**Conclusion:**

KHF exerts anti-inflammatory and anti-fibrotic effects in AE-IPF, partly by modulating GLUT1-mediated glycolysis and regulating the GLUT1/HIF-1α axis, with rutin as a key bioactive component. These findings support the clinical application of KHF and highlight GLUT1-centered metabolic pathways as potential therapeutic targets.

## 1 Introduction

Idiopathic pulmonary fibrosis (IPF) is a progressive interstitial lung disease with a poor prognosis, in which persistent fibrotic remodeling of the lung parenchyma gradually compromises respiratory function. Acute exacerbation of IPF (AE-IPF) represents the most severe clinical manifestation of this disease, characterized by sudden or subacute respiratory deterioration occurring on the background of chronic fibrosis. Patients with AE-IPF face extremely high short-term mortality, and effective therapeutic options remain limited (1). From a pathological perspective, AE-IPF is commonly accompanied by diffuse alveolar damage and rapidly progressive fibrosis, processes that are closely linked to dysregulated inflammatory responses (2). Cytokine profiling of bronchoalveolar lavage fluid (Balf) from patients with AE−IPF revealed prominent macrophage activation, characterized by marked upregulation of pro−inflammatory chemokines (such as interleukin−8 and C−X−C motif chemokine ligand 1) as well as M2 macrophage markers, indicating that macrophage polarization imbalance is closely associated with the rapid progression of the disease (3). These observations imply that excessive macrophage activation and polarization imbalance may contribute to disease exacerbation, making macrophage-mediated inflammation a potential therapeutic target.

In recent years, studies in immunometabolism have highlighted a close association between macrophage activation and metabolic reprogramming, particularly enhanced glycolysis (4). Upon exposure to inflammatory stimuli, macrophages exhibit a metabolic shift toward increased glycolytic flux, even under normoxic conditions. This shift provides rapid energy supply to support cytokine production, migration, and phagocytic activity (5). Beyond energy generation, glycolytic intermediates have been shown to participate in intracellular signaling and transcriptional regulation, thereby reinforcing inflammatory phenotypes (6). Key glycolytic regulators, including glucose transporter 1 (GLUT1, encoded by SLC2A1) and hexokinase 2 (HK2), are consistently upregulated during macrophage activation and are required to sustain high glycolytic activity (7). Moreover, alterations in glycolytic metabolism influence immune signaling pathways and macrophage functional states (8). Together, these findings indicate that dysregulated glycolysis represents an important pathological feature of inflammatory lung diseases, although its therapeutic exploitation in AE-IPF remains limited.

Within the framework of traditional Chinese medicine (TCM), AE-IPF is classified under the syndromes of Feibi (lung obstruction) and Feiwei (lung wilting), with disease progression attributed to phlegm-heat accumulation, qi deficiency, and blood stasis (9, 10). Based on these concepts, Professor Jiansheng Li developed the Kangxianhuanji Formula (KHF), which follows the therapeutic principles of resolving phlegm, activating blood circulation, clearing heat and detoxifying, and tonifying the lung and kidney. KHF was derived from modifications of Bufei Decoction and Houpo Sanwu Decoction and formulated according to the Jun–Chen–Zuo–Shi theory. Clinical studies have reported that KHF, when used as an adjunct to conventional therapy, reduces treatment failure rates, shortens hospitalization, alleviates dyspnea, and improves quality of life in patients with AE-IPF (10). KHF consists of multiple medicinal herbs, including Fritillaria thunbergii Miq., Epimedium brevicornum Maxim., Paeonia suffruticosa Andrews, Curcuma longa L., Panax ginseng C. A. Mey., and Glycyrrhiza uralensis Fisch., all of which are documented in the Pharmacopoeia of the People’s Republic of China (2025 edition) and have long been used for respiratory disorders. Experimental studies have provided pharmacological evidence supporting the anti-inflammatory and anti-fibrotic effects of these herbs. For instance, Fritillaria thunbergii suppresses nitric oxide (NO), tumor necrosis factor-alpha (TNF-α), and interleukin-6 (IL-6) production in lipopolysaccharide (LPS)-stimulated macrophages and inhibits fibroblast glycolysis through the phosphatidylinositol 3-kinase (PI3K)/protein kinase B (Akt)/PFKFB3 pathway (11, 12). Paeonia suffruticosa reduces collagen deposition and downregulates transforming growth factor-beta 1 (TGF-β1), collagen type I alpha 1 chain (COL1A1), and alpha-smooth muscle actin (α-SMA) expression in pulmonary fibrosis models (13), while Epimedium brevicornum attenuates bleomycin-induced fibrosis by inhibiting inflammatory responses, collagen synthesis, and yes-associated protein signaling (14). In addition, Panax ginseng has been shown to modulate abnormal glycolysis via the PI3K/Akt/mechanistic target of rapamycin (mTOR)/hypoxia-inducible factor 1-alpha (HIF-1α) pathway (15), and Curcuma longa suppresses glycolytic activity through 5′-AMP-activated protein kinase (AMPK)-dependent mechanisms (16). Collectively, these findings suggest that the constituent herbs of KHF possess both anti-inflammatory and glycolysis-regulating properties, supporting its potential role in modulating immunometabolic pathways in AE-IPF.

Despite these advances, the molecular mechanisms underlying the therapeutic actions of KHF in AE-IPF remain incompletely understood. In particular, whether KHF exerts its anti-inflammatory and anti-fibrotic effects through regulation of macrophage glycolytic metabolism has not been clearly addressed, and the key bioactive constituents responsible for these effects remain to be identified. To address these gaps, the present study systematically evaluated the effects of KHF in AE-IPF models *in vivo* and *in vitro* and explored its underlying mechanisms. An integrated strategy combining network pharmacology, DARTS and proteomics was employed to identify potential targets and pathways, validate direct protein interactions, and characterize global protein alterations *in vivo*. These complementary approaches enabled cross-validation across multiple levels, including computational prediction, molecular interaction, and functional regulation. Through this integrative analysis, the study aimed to identify key targets involved in macrophage metabolic reprogramming and to elucidate the molecular mechanisms underlying the therapeutic effects of KHF. In addition, key bioactive constituents responsible for its pharmacological activity were further characterized, providing a mechanistic basis for its clinical application in AE-IPF.

## 2 Materials and methods

### 2.1 Animals and ethics statement

A total of fifty specific pathogen-free (SPF) male C57BL/6J mice (6–8 weeks old, 21 ± 2 g) were obtained from SPF (Beijing) Biotechnology Co., Ltd. The animals were housed in the Animal Experimental Center of Henan University of Chinese Medicine under controlled environmental conditions (temperature 23 ± 2 °C, relative humidity 50 – 65%, 12 h light/dark cycle). All experimental procedures were conducted in accordance with institutional ethical guidelines and approved by the Animal Ethics Committee of Henan University of Chinese Medicine (Approval No. IACUC-202311019). The animal facility is licensed under SYXK (Yu) 2021-0015. After a 7-day acclimatization period, all animals were used for the subsequent experimental procedures.

### 2.2 AE-IPF model establishment and drug administration

Fifty C57BL/6J mice were randomly assigned into five groups: blank control group, model group, KHF low-dose group, KHF medium-dose group, and prednisone acetate group. Pulmonary fibrosis was induced by intratracheal instillation of bleomycin. Mice in the blank control group received sterile saline intratracheally on days 0, 14, 28, and 56. Mice in the model group, KHF low-dose group, KHF medium-dose group, and prednisone group were administered bleomycin at a low dose (3 mg/kg) intratracheally on days 0, 14, and 28, followed by a high dose (5 mg/kg) on day 56. From day 57, mice in the KHF low- and medium-dose groups were intragastrically administered KHF at doses of 9.3 g/kg/day and 18.6 g/kg/day, respectively, Notably, the medium dose (18.6 g/kg/day) was calculated based on human equivalent dose conversion and represents the clinically relevant dosage. A high-dose group was not included, as the present study primarily focused on evaluating therapeutic efficacy within a clinically relevant dosing range rather than establishing a full dose–response relationship. The prednisone group received prednisone acetate at 4.55 mg/kg/day. All treatments were given once daily for 14 consecutive days. Body weight was monitored every 3 days, and drug dosages were adjusted accordingly. On day 70, mice were sacrificed for sample collection. Drug dosages for mice were calculated based on human equivalent dose conversion using the body surface area normalization method:D_mouse_ = D_human_ ×(HI_mouse_/HI_human_) × (W_human/_W_mouse_)^2/3^ (17). where D represents the dose (mg/kg), HI is the body surface area coefficient, and W is body weight (kg).

### 2.3 Anesthesia, blood collection, and euthanasia

Mice were anesthetized with isoflurane inhalation (3–4% for induction and 1.5–2% for maintenance, delivered via a precision vaporizer). The depth of anesthesia was assessed by the absence of the pedal withdrawal reflex and a stable respiratory rate. Blood samples were collected via the orbital venous sinus under deep anesthesia. After blood collection, mice were euthanized by cervical dislocation while still under deep anesthesia.

### 2.4 Drugs, reagents, and preparation of KHF

The KHF was composed of the following crude herbs: *Gynostemma pentaphyllum* (Thunb.) Makino (Jiaogulan, 15g, Batch No.111710195, Anhui Meiyu Pharmaceutical Co., Ltd, Anhui, China), *Epimedium brevicornum* Maxim. (Yinyanghuo, 9g, Batch No.C221009181, Chengdu Runde Pharmaceutical Co., Ltd., Chengdu, China), *Fritillaria thunbergii* Miq. (Zhebeimu, 9g, Batch No.22100102, Zhengzhou Ruilong Pharmaceutical Co., Ltd, Henan, China), *Paeonia suffruticosa* Andrews (Mudanpi, 12g, Batch No.22100101, Zhengzhou Ruilong Pharmaceutical Co., Ltd, Henan, China), *Curcuma longa* L. (Jianghuang, 9g, Batch No.211101, Anhui Shouxin Traditional Chinese Medicine Technology Co., Ltd, Anhui, China), *Coix lacryma-jobi* L. (Yiyiren, 15g, Batch No.22090101, Zhengzhou Ruilong Pharmaceutical Co., Ltd, Henan, China), *Panax ginseng* C.A.Mey. (Renshen, 6g, Batch No.22050101, Zhengzhou Ruilong Pharmaceutical Co., Ltd, Henan, China), *Glycyrrhiza uralensis* Fisch.(Gancao, 12g, Batch No.22060103, Zhengzhou Ruilong Pharmaceutical Co., Ltd, Henan, China), among others. All botanical names were verified with the World Flora Online database (accessed on 22 October 2025). The herbal materials were authenticated by Associate Professor Yu Fu from the School of Pharmacy, Henan University of Chinese Medicine, and complied with the quality standards specified in the Chinese Pharmacopoeia (2020 edition). KHF was prepared from its constituent herbal materials according to standardized traditional decoction procedures. Individual herbs were pretreated or decocted in accordance with their conventional processing requirements to ensure appropriate extraction of active constituents. Briefly, the crude herbs were mixed and extracted with purified water and decocted under boiling conditions until the solvent volume was substantially reduced to obtain a concentrated extract. The extract was subsequently freeze-dried to yield a lyophilized powder. The extraction yield was calculated based on the ratio of the freeze-dried powder to the initial weight of crude herbal materials, with 1 g of KHF powder equivalent to 2.705 g of crude drugs. The powder was aliquoted, sealed, and stored at 4 °C until use. Prior to administration, KHF powder was reconstituted in sterile water. All preparations were performed under consistent conditions to ensure batch-to-batch reproducibility. Prednisone acetate tablets (Batch No. H34021846; Jin Taiyang, Anhui, China) and bleomycin sulfate (Batch No. B802467; Macklin, Shanghai, China) were used as reference drugs. Isoflurane (Cat. No. R510-22-10; RWD Life Science, Shenzhen, China), Hematoxylin-eosin staining kit and Masson’s trichrome staining kit (Lot No. 20180603 and G1364; Solarbio, Beijing, China), hydroxyproline assay kit (Lot No. A030-2-1; Nanjing Jiancheng, Nanjing, China), Bradford protein assay kit (Lot No. P0006; Beyotime, Shanghai, China), and streptavidin–biotin complex–peroxidase (SABC-POD) (Rabbit IgG) immunohistochemistry kit (Lot No. SA2002; Bioss, Wuhan, China) were used for histological and biochemical assays. Additional reagents included dimethyl sulfoxide (DMSO) (Lot No. S26063; YuanYe, Shanghai, China), fetal bovine serum (premium grade), penicillin-streptomycin solution (100 ×), Dulbecco’s Modified Eagle Medium (DMEM) high-glucose medium, and One-step Sodium dodecyl sulfate–polyacrylamide gel electrophoresis (SDS-PAGE) gel preparation kit (Lot Nos. G8002, G4003, G4511, G2177; Servicebio, Wuhan, China). LPS (CAS: 93572-42-0; Merck, Shanghai, China) and Cell Viability Assay (CCK-8) kit (Lot No. K1018; APExBIO, Houston, USA) were used for cell experiments. HiScript IV All-in-One Ultra RT SuperMix for quantitative reverse transcription polymerase chain reaction (qRT-PCR) (Lot No. R433-01; Vazyme, Nanjing, China) was used for reverse transcription. Primary antibodies against high mobility group box 1 (HMGB1, 10829-1-AP), IL-6 (26404-1-AP), and TNF-α, (17590-1-AP) were obtained from Proteintech (Wuhan, China). mammalian protein extraction reagent (M-PER) lysis buffer (Lot No. 78501; Thermo Fisher Scientific, USA), bicinchoninic acid (BCA) protein assay kits (Lot No. ZJ102, Epizyme, Shanghai, China), BeyoBlue Coomassie staining solution (Lot No. P0017F, Beyotime, Shanghai, China), L-Lactate Assay Kit with WST-8 (Lot No. A544A251231, Beyotime, Shanghai, China), streptavidin (Lot No. 10165921001; Merck, Shanghai, China), protease inhibitor cocktail (Lot No. 78425; Thermo, USA), urea (Lot No. U820349; Macklin, Shanghai, China), dithiothreitol (Lot No. 43815; Merck, Shanghai, China), ammonium bicarbonate (Lot No. A800859; Macklin, Shanghai, China), trypsin (Lot No. V5111; Promega, Madison, USA), and peptide quantitation kit (Lot No. 23275; Thermo, USA) were applied in proteomic analyses. Chromatographic-grade acetonitrile and methanol (Lot Nos. 1060074008, 1000304008; Merck, Shanghai, China) were used for LC–MS/MS analysis. Rutin (Lot No.A0103;Manster, Chengdu, China).

### 2.5 Histological evaluation

Left lung tissues were fixed in 10% neutral buffered formalin for 72 h, embedded in paraffin, and sectioned at 4 μm thickness. Sections were stained with HE and Masson’s trichrome following standard protocols. Histopathological changes were examined under a light microscope. Inflammation was scored using the Szapiel grading system (18), and fibrosis was evaluated using the Ashcroft scoring system (19). All assessments were performed in a blinded manner.

### 2.6 Immunofluorescence staining

Left lung tissues were paraffin-embedded and sectioned at 4 μm thickness. Sections were deparaffinized and subjected to antigen retrieval using sodium citrate buffer. After cooling, sections were incubated with 5% bovine serum albumin at 37 °C for 30 min to block non-specific binding. Sections were then incubated with primary antibody against surfactant protein C (SPC) at 4 °C overnight. After washing, sections were incubated with fluorescently labeled secondary antibodies at room temperature for 1 h in the dark. Nuclei were counterstained with DAPI. Fluorescence images were captured under a fluorescence microscope. The expression level of SPC was quantified using ImagePro Plus 6.0 software after image acquisition and analysis.

### 2.7 Enzyme-linked immunosorbent assay

Approximately 10 mg of the right lung tissue was homogenized in 90 μL phosphate-buffered saline (PBS) using a high-speed refrigerated tissue homogenizer. The homogenates were centrifuged, and the supernatants were collected. Protein concentrations were determined using the BCA protein assay, and all samples were diluted with PBS to equal concentrations. Cytokine levels were determined according to the manufacturer’s instructions of the BD ELISA kits. Briefly, 96-well plates were coated overnight with capture antibodies, followed by washing, blocking, sample addition, incubation with detection antibodies, and substrate development. The absorbance values were measured at 450 nm with correction at 570 nm using a microplate reader. A standard curve was generated to calculate the concentrations of interleukin-1 beta (IL-1β), IL-6, and TNF-α.

### 2.8 Immunohistochemistry

Left lung tissues were paraffin-embedded and sectioned at 4 μm thickness. Sections were deparaffinized and subjected to antigen retrieval using sodium citrate buffer in a water bath. Endogenous peroxidase activity was blocked with 3% hydrogen peroxide, followed by incubation with 5% goat serum at 37 °C for 30 min to block non-specific binding. Sections were then incubated with primary antibodies against HMGB1, IL-6, and TNF-α at 4 °C for 10–12 h. After washing, sections were incubated with biotinylated goat anti-rabbit IgG at room temperature for 20 min, washed, and then incubated with SABC complex for 30 min at room temperature. Visualization was performed using 3,3′-diaminobenzidine (DAB) substrate; the reaction was terminated by immersing the slides in running water once the tissue turned light brown. Nuclei were counterstained with 0.1% Mayer’s hematoxylin, and sections were dehydrated and mounted. Protein expression levels of HMGB1, IL-6, and TNF-α were quantified using Image-Pro Plus 6.0 software after microscopic observation and imaging.

### 2.9 Network pharmacology analysis

Active compounds and corresponding targets of KHF were retrieved from the TCMSP database using oral bioavailability ≥ 30% and drug-likeness ≥ 0.18 as screening criteria (20). Disease-related targets of AE-IPF were collected from GeneCards, OMIM, DisGeNET, and DrugBank databases. Overlapping targets were identified and visualized using Venn diagrams. protein–protein interaction (PPI) networks were constructed using the STRING database and analyzed with Cytoscape. Gene Ontology (GO) and Kyoto Encyclopedia of Genes and Genomes (KEGG) enrichment analyses were performed using DAVID with P < 0.05 as the significance threshold.

### 2.10 DARTS-based target identification

RAW264.7 cells were cultured in DMEM supplemented with 10% fetal bovine serum for 24 h. Cells were collected, washed with PBS, and lysed using M-PER lysis buffer with sonication, followed by incubation on ice for 10 min. The lysates were centrifuged, and the supernatants were collected to prepare total cellular protein using 1 × TNC buffer. Protein samples were divided into two groups and incubated with either KHF extract (experimental group) or DMSO (control group). After incubation, samples were subjected to gradient proteolysis with varying concentrations of pronase to induce partial protein degradation. Proteins were separated by SDS-PAGE and visualized using Coomassie Brilliant Blue staining. Differential bands showing resistance to degradation in the KHF-treated group were excised, digested with trypsin, and the resulting peptides were extracted. Peptide samples were dried by centrifugation and reconstituted in 10% formic acid for analysis using a NanoLC-Q-Exactive mass spectrometry system. Mass spectrometry data were analyzed with a fold change >1.5 as the cutoff to identify candidate proteins with strong binding affinity to KHF. GO and KEGG pathway enrichment analyses were performed on the candidate proteins (P < 0.05). Finally, the targets identified by DARTS were intersected with core targets predicted by prior network pharmacology analysis to pinpoint key targets. GraphPad Prism software was used to visualize peptide counts, sequence coverage, and relative abundance, providing a comprehensive assessment of the reliability of these proteins as potential targets of KHF.

### 2.11 Label-free quantitative proteomics

Lung tissue samples were homogenized in RIPA lysis buffer containing protease inhibitors to extract total proteins. Proteins were reduced with Dithiothreitol and alkylated with iodoacetamide, followed by overnight digestion with trypsin at 37 °C. The digestion was terminated with Trifluoroacetic acid, and peptides were desalted and purified using C18 columns. Peptide concentrations were quantified prior to analysis. Peptides were separated on a 75 μm × 20 cm C18 column using a NanoLC-Q Exactive system with a gradient elution from 0.1% formic acid to 80% acetonitrile. Mass spectrometry acquisition parameters were set as follows: MS1 resolution 70,000, MS2 resolution 17,500, HCD fragmentation mode, collision energy 27 eV. Proteins were identified using Proteome Discoverer 2.5 with database searching. Peptide intensities were normalized using the mean total peptide intensity method. Differentially expressed proteins were defined as those with P < 0.05 and fold change > 1.2 or < 0.83.

### 2.12 Ultra-high performance liquid chromatography-mass spectrometry identification of KHF components

One milliliter of KHF solution was mixed with 3 mL ethanol, vortexed for 3 min, sonicated in an ice bath for 10 min, and incubated at room temperature for 12 h. Samples were centrifuged at 7,000 g for 20 min at 4 °C, and the supernatant was collected. Ethanol was removed under a nitrogen stream to obtain the purified extract. Before mass spectrometry analysis, the extract was reconstituted in ultrapure water to 1 mL, filtered through a 0.22 μm membrane, and 10 μL was injected for analysis, with technical triplicates for each sample. Chromatographic separation was performed using a SHIMADZU-LC30 UHPLC system with an ACQUITY UPLC^®^ HSS T3 column (2.1 × 100 mm, 1.8 μm; Waters, Milford, MA, USA) at 40 °C, flow rate 0.3 mL/min. Mobile phases were 0.1% formic acid in water (A) and 0.1% formic acid in acetonitrile (B). Gradient elution was as follows: 0–2 min, 0% B; 2–4 min, 25% B; 4–22 min, 25–100% B; 22–25 min, 100% B; 25–25.1 min, 100–0% B; 25.1–30 min, 0% B. Mass spectrometry was performed on a QE Plus system (Thermo Scientific) using a heated electrospray ionization source in both positive and negative modes. Raw MS data were processed with MS-DIAL for peak alignment, retention time correction, and peak area extraction. Metabolites were identified with mass tolerances of < 0.01 Da for MS1 and < 0.02 Da for MS2, and an MS2 matching score > 70%. A total of 807 chemical constituents were identified by LC–MS/MS analysis. The top 30 compounds with the highest matching scores were selected for further analysis.

### 2.13 Preparation of concentrated KHF extract for cell experiments

Before cell treatment, the aqueous extract of KHF was loaded onto a macroporous resin column to allow adsorption of active components. Sequential elution was performed using ultrapure water and a gradient of industrial ethanol concentrations (0%, 10%, 30%, 60%, 90%, and 100%), and the eluates were collected. All fractions were combined and concentrated to dryness using a rotary evaporator to obtain the full KHF extract powder. The powder was dissolved in DMSO and stored at 4 °C in the dark until use.

### 2.14 Cell culture, grouping, and treatment

RAW264.7 murine macrophages were cultured in DMEM supplemented with 10% fetal bovine serum and 1% penicillin–streptomycin under standard conditions (37 °C, 5% CO_2_, and humidified atmosphere). The culture medium was replaced daily, and cell morphology and growth status were monitored regularly. Cells were passaged at a ratio of 1:3–1:4 when reaching approximately 80% confluence. Cells in the logarithmic growth phase were used for all experiments. For experimental treatments, RAW264.7 cells were seeded in 12-well plates at a density of 3 × 10^5^ cells per well. Cells were divided into the following groups: control group, LPS group (500 ng/mL), KHF-treated groups (50, 100, and 200 μg/mL), Rutin-treated groups (50, 100, and 200 μM), GLUT1 inhibitor group (STF-31, 5 μM), KHF plus STF-31 group (100 μg/mL KHF + 5 μM STF-31), Rutin plus STF-31 group (100 μM Rutin + 5 μM STF-31), and prednisolone group (5 μM). Except for the control group, all groups were treated with LPS (500 ng/mL) to induce inflammation. After 12 h of incubation, cells were collected for subsequent analyses. Drug concentrations were determined based on preliminary experimental results.

### 2.15 CCK-8

RAW264.7 cells were seeded in 96-well plates at a density of 1 × 10^4^ cells per well and cultured for 24 h to allow adherence. The medium was then replaced with fresh medium containing different concentrations of KHF (0, 25, 50, 100, and 200 μg/mL), followed by incubation for another 24 h. Subsequently, 10 μL of CCK-8 solution was added to each well, and the cells were incubated at 37 °C for 2 h. After incubation, absorbance was measured at 450 nm using a microplate reader. Cell viability was calculated as follows: Cell viability (%) = Atreated/Acontrol × 100%.Lactate Assay.

RAW264.7 cells were cultured and treated according to the experimental design. After treatment, the culture supernatants were collected and centrifuged to remove cell debris.

### 2.16 Lactate assay

Lactate levels in the cell culture supernatants were measured using a commercial lactate assay kit according to the manufacturer’s instructions. Briefly, samples and standards were added to a 96-well plate, followed by the addition of the working reagent. After incubation at room temperature for 30 min, absorbance was measured at the indicated wavelength using a microplate reader. Lactate concentrations were calculated based on the standard curve. All measurements were performed in triplicate.

### 2.17 Cellular thermal shift assay

RAW264.7 cells were cultured and treated with KHF or vehicle control according to the experimental design. After treatment, cells were harvested, washed with PBS, and resuspended in lysis buffer supplemented with protease inhibitors. Cell suspensions were aliquoted into PCR tubes and heated at a series of temperatures (37, 42, 47, 52, and 57 °C) for 3 min, followed by cooling to room temperature for 3 min. The samples were then subjected to repeated freeze–thaw cycles to ensure complete lysis and subsequently centrifuged at 12,000 × g for 20 min at 4 °C to separate soluble protein fractions. The supernatants were collected, and protein levels were analyzed by western blotting to detect the target protein. Band intensities were quantified using ImageJ software, and the relative protein stability was evaluated by comparing the remaining soluble protein at different temperatures among groups.

### 2.18 qRT-PCR

The expression levels of IL-1β, IL-6, TNF-α, SLC2A1, HIF-1α, and HK2 in RAW264.7 cells were determined using qRT-PCR. Total RNA was extracted from the cells using 1 mL of TRIzol reagent according to the manufacturer’s protocol. The RNA pellet was dissolved in 31 μL of DEPC-treated water and incubated at room temperature for 5 min, after which RNA concentration and purity were measured. RNA samples were then diluted to a uniform concentration and reverse-transcribed into cDNA. According to the SYBR Green detection system requirements, primers and diluted cDNA samples were prepared and loaded into a 384-well plate. Amplification was performed using a real-time PCR instrument under standard cycling conditions. All primer sequences are listed in **Table 1**.

**Table 1:** Primer sequence.

Gene	Primer sequence (5'-3')	bp
IL-1β	F: GAAATGCCACCTTTTGACAGTG R: TGGATGCTCTCATCAGGACAG	116
IL-6	F: CTGCAAGAGACTTCCATCCAG R: AGTGGTATAGACAGGTCTGTTGG	131
TNF-α	F: CAGGCGGTGCCTATGTCTC R: CGATCACCCCGAAGTTCAGTAG	173
SLC2A1	F: GCAGTTCGGCTATAACACTGG R: GCGGTGGTTCCATGTTTGATTG	173
HIF-1α	F: ACCTTCATCGGAAACTCCAAAG R: CTGTTAGGCTGGGAAAAGTTAGG	182
HK2	F: TGATCGCCTGCTTATTCACGG R: AACCGCCTAGAAATCTCCAGA	152
*β*-actin	F: GTGACGTTGACATCCGTAAAGA R: GCCGGACTCATCGTACTCC	245
GAPDH	F: AGGTCGGTGTGAACGGATTTG R: GGGGTCGTTGATGGCAACA	95

### 2.19 Statistical analysis

Statistical analyses were performed using GraphPad Prism (version 10.1.2). Data are presented as mean ± standard deviation (SD). Differences among multiple groups were analyzed using one-way analysis of variance (ANOVA) followed by Dunnett’s *post hoc* test for multiple comparisons. When data did not meet the assumptions of normality or homogeneity of variance, the Kruskal–Wallis test was applied. For *in vitro* experiments, data represent at least three independent biological replicates, each with technical triplicates. For *in vivo* experiments, the number of animals per group is indicated in the corresponding figure legends. A value of *P* < 0.05 was considered statistically significant.

## 3 Results

### 3.1 KHF ameliorates inflammation and fibrosis in bleomycin-induced AE-IPF mice and suppresses macrophage activation *in vitro*

**Figure 1:**
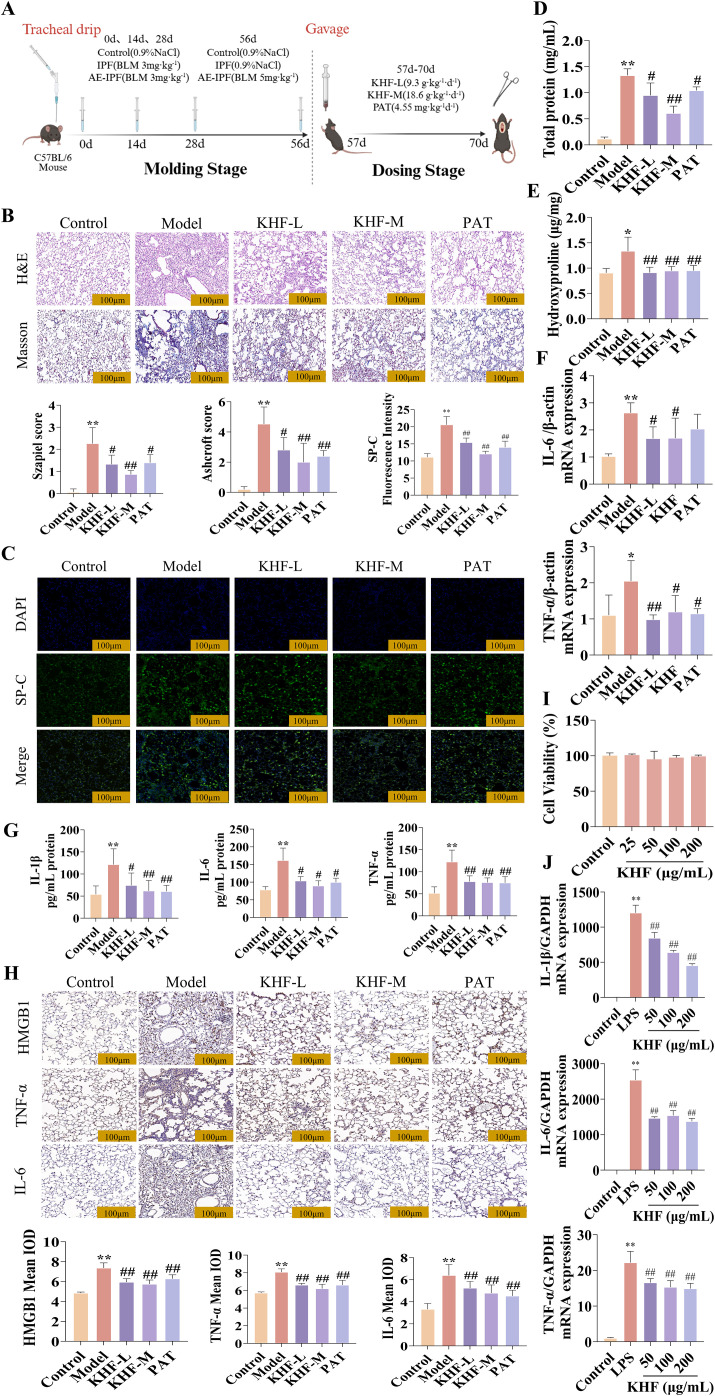
KHF alleviates bleomycin-induced AE-IPF by suppressing macrophage-mediated inflammatory responses. **(A)** Schematic illustration of the modeling and drug administration protocol for acute exacerbation of pulmonary fibrosis in mice. **(B)** Representative histopathological images of lung tissue (HE and Masson’s trichrome staining, ×200). **(C)** Immunofluorescence staining of SPC in lung tissue (×200). **(D)** Total protein concentration in Balf. **(E)** Hydroxyproline content in lung tissue. **(F)** Relative mRNA expression levels of inflammatory mediators in lung tissue. **(G)** Protein levels of inflammatory mediators in lung tissue measured by ELISA. **(H)** Immunohistochemical staining of inflammatory mediators in lung tissue. **(I)** Cell viability of RAW264.7 macrophages treated with different concentrations of KHF. **(J)** Relative mRNA expression levels of inflammatory mediators in RAW264.7 cells measured by qRT-PCR. Data are presented as mean ± SD; *n* = 6 per group. *P < 0.05, **P < 0.01 vs. model group; ^#^*P* < 0.05, ^##^*P* < 0.01 vs. KHF group.

To evaluate the therapeutic effects of KHF, a bleomycin-induced AE-IPF mouse model was established, and the experimental workflow is illustrated in **Figure 1A**. Histopathological examination using HE and Masson’s trichrome staining showed prominent inflammatory cell infiltration, alveolar structure disruption, and collagen deposition, which were markedly alleviated by KHF treatment (**Figure 1B**). Immunofluorescence staining of surfactant protein C (SPC) revealed increased expression, indicating abnormal activation or proliferation of alveolar type II epithelial cells, while KHF treatment reduced SPC fluorescence intensity, suggesting improved epithelial homeostasis (**Figure 1C**), BALF analysis demonstrated elevated protein leakage, which was attenuated by KHF (**Figure 1D**). Hydroxyproline content, reflecting collagen accumulation, was reduced following KHF administration (**Figure 1E**). AAt the molecular level, KHF suppressed the mRNA expression of pro-inflammatory cytokines, including IL-6 and TNF-α (**Figure 1F**). ELISA results showed reduced protein levels of IL-1β, IL-6, and TNF-α (**Figure 1G**). Immunohistochemical staining further demonstrated decreased expression of IL-1β, TNF-α, and high mobility group box 1 (HMGB1) (**Figure 1H**). *In vitro* experiments in RAW264.7 macrophages showed no significant cytotoxicity of KHF within the tested concentration range (**Figure 1I**). Under these conditions, KHF inhibited LPS-induced mRNA expression of IL-1β, IL-6, and TNF-α in a dose-dependent manner (**Figure 1J**). Collectively, these findings demonstrate that KHF alleviates lung injury, inflammation, and fibrosis in AE-IPF, accompanied by suppression of macrophage-mediated inflammatory responses.

### 3.2 Network pharmacology analysis identifies key targets of KHF

**Figure 2:**
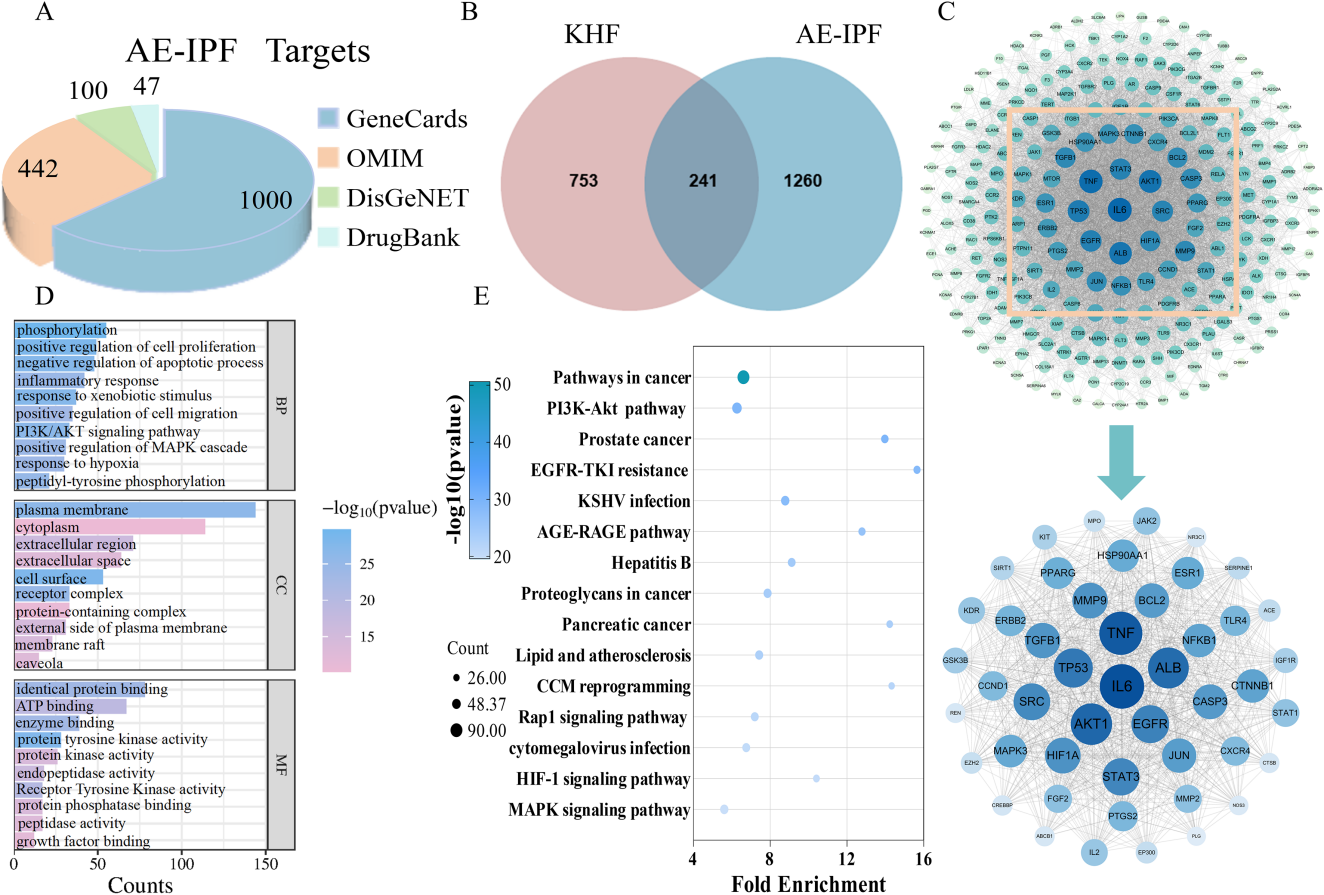
Network pharmacology analysis of potential targets of KHF in AE-IPF. **(A)** Summary of AE-IPF-related targets retrieved from public databases. **(B)** Venn diagram of overlapping targets between KHF and AE-IPF. **(C)** PPI network of overlapping targets. **(D)** GO enrichment analysis. **(E)** KEGG pathway enrichment analysis.

To systematically explore the potential molecular mechanisms underlying the effects of KHF, a network pharmacology approach was employed. A total of 1,501 AE-IPF-related targets were collected from multiple disease databases, while 994 putative targets were predicted from the active components of KHF. Intersection analysis identified 241 shared targets between KHF and AE-IPF (**Figures 2A, B**). PPI network analysis revealed a tightly connected target network (**Figure 2C**). GO functional annotation and KEGG pathway enrichment analyses indicated that these targets were primarily involved in biological processes related to phosphorylation, cell proliferation, and metabolic regulation. Notably, several signaling pathways associated with energy metabolism and inflammation, including the PI3K–Akt and HIF-1α signaling pathways, were significantly enriched (**Figures 2D, E**). These findings suggest that KHF may exert its therapeutic effects through coordinated regulation of inflammatory responses and metabolic reprogramming in AE-IPF.

### 3.3 DARTS identifies GLUT1 as a direct target of KHF

**Figure 3:**
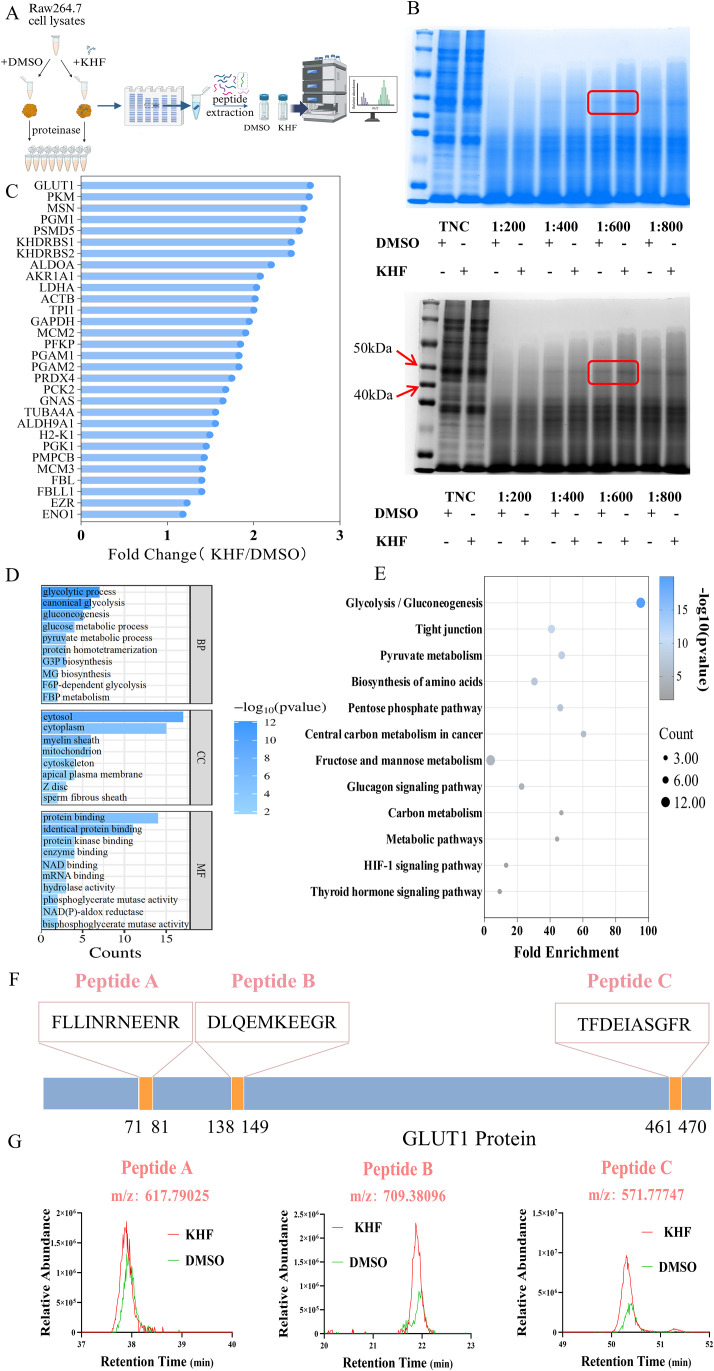
Identification of key protein targets of KHF using DARTS and proteomic analysis. **(A)** Schematic workflow of DARTS-based target identification. **(B)** Representative SDS–PAGE gel showing protease protection at different pronase concentrations. **(C)** Differentially expressed proteins between KHF and DMSO groups. **(D)** GO enrichment analysis of differentially expressed proteins. **(E)** KEGG pathway enrichment analysis. **(F)** LC–MS/MS identification of peptide fragments corresponding to GLUT1. **(G)** Relative abundance of GLUT1 peptides in KHF and DMSO groups.

DARTS is a technique that identifies drug targets based on changes in protein stability, allowing detection of direct binding targets by comparing protein degradation under drug-treated and control conditions (21) (**Figure 3A**). Using DARTS to capture proteins directly interacting with KHF, a significant difference in protein bands was observed between the KHF-treated and control groups (**Figure 3B**). Subsequent mass spectrometry analysis identified 30 differential proteins (**Figure 3C**). Enrichment analysis of all captured proteins indicated that they were primarily involved in glycolysis/gluconeogenesis and HIF-1α signaling pathways (**Figures 3D, E**). Notably, GLUT1 emerged as the most prominent differential protein and was also identified as a shared target in both network pharmacology and DARTS analyses. LC-MS/MS analysis of GLUT1 detected three peptide fragments, all of which showed significantly higher relative abundance in the KHF-treated group compared to the DMSO control (**Figures 3F, G**). As the primary glucose transporter on the cell membrane, GLUT1 facilitates transmembrane glucose uptake, providing essential substrates for glycolysis. Previous studies indicate that GLUT1-mediated glucose uptake represents a rate-limiting step in glycolysis, and its expression directly affects intracellular glucose availability and glycolytic flux (22). Taken together, these results suggest that KHF may exert its pharmacological effects by targeting GLUT1-mediated glycolysis.

### 3.4 Proteomic profiling reveals suppression of glycolysis by KHF in AE-IPF lungs

**Figure 4:**
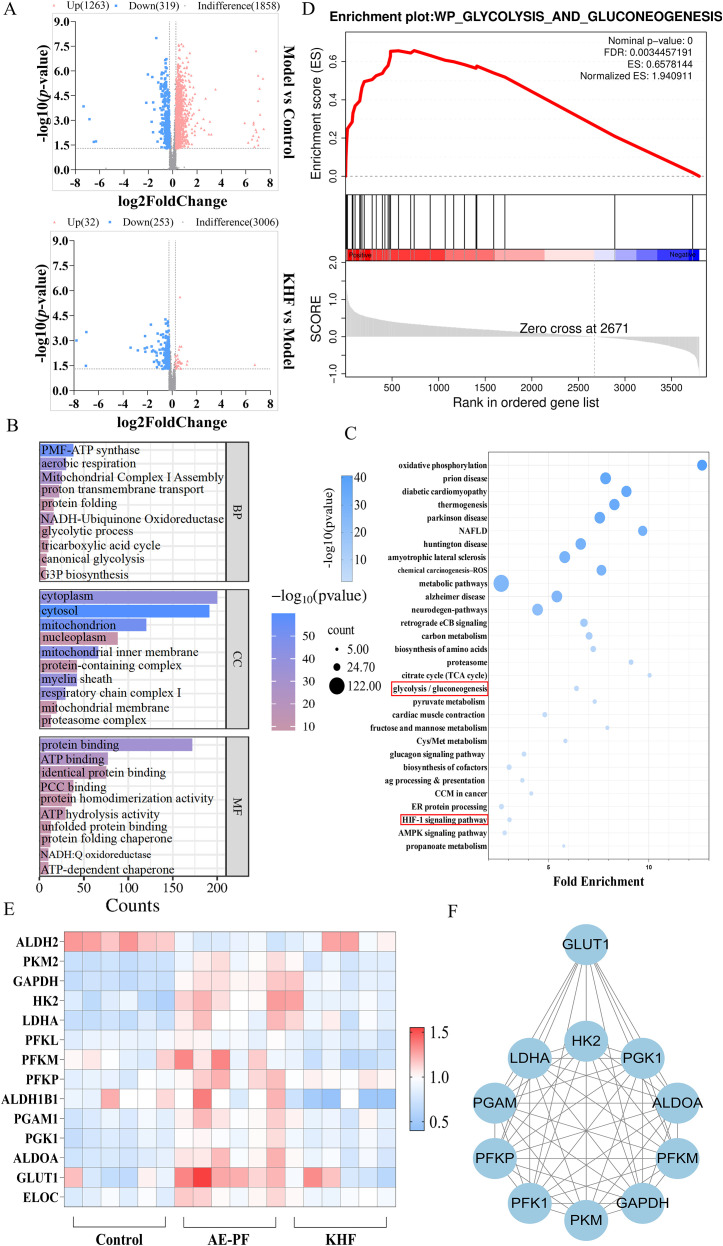
Proteomic profiling reveals that KHF regulates glycolysis-related pathways in lung tissue. **(A)** Volcano plot of differentially expressed proteins. **(B)** GO enrichment analysis of differentially expressed proteins. **(C)** KEGG pathway enrichment analysis. **(D)** GSEA indicating enrichment of the glycolysis/gluconeogenesis pathway. **(E)** Heatmap of glycolysis-related proteins among the differentially expressed proteins. **(F)** Network representation of GLUT1 and associated glycolytic proteins.

To further investigate the mechanism of KHF, proteomic analysis was performed to examine its impact on protein expression in the lungs of mice and to validate its inhibitory effect on glycolysis. Compared with the control group, the AE-IPF model group exhibited 1,263 upregulated proteins and 319 downregulated proteins. In contrast, treatment with KHF resulted in 253 downregulated proteins and 32 upregulated proteins compared with the model group (**Figure 4A**). GO and KEGG enrichment analyses indicated that the therapeutic effects of KHF were closely associated with systemic inhibition of the glycolysis/gluconeogenesis pathway (**Figures 4B, C**), which was further supported by gene set enrichment analysis (GSEA) analysis (**Figure 4D**). Heatmap analysis of differentially expressed proteins revealed that KHF suppressed multiple key proteins within this pathway, including GLUT1. PPI network analysis of these differential proteins further highlighted GLUT1 as a critical target mediating KHF’s regulation of glycolysis (**Figures 4E, F**). Collectively, these results confirm that KHF ameliorates AE-IPF pathology by modulating glycolysis-centered metabolic pathways, with its effects closely associated with GLUT1 expression.

### 3.5 KHF inhibits HIF-1α–dependent glycolysis via targeting GLUT1 in macrophages

**Figure 5:**
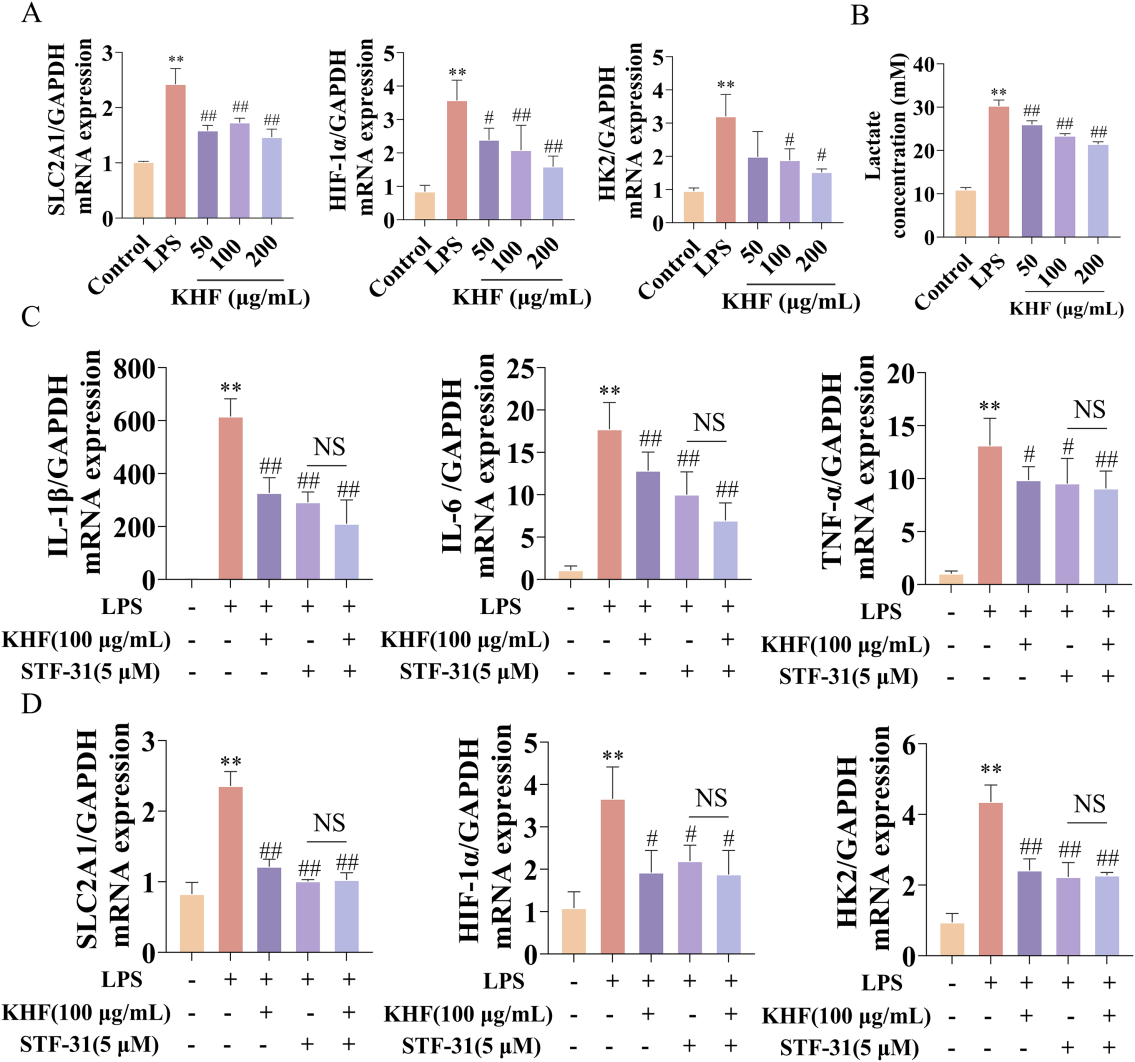
KHF suppresses HIF-1α-mediated glycolysis by targeting GLUT1 in macrophages. **(A)** Relative mRNA expression levels of SLC2A1 (GLUT1), HIF-1α, and HK2 in RAW264.7 cells under LPS stimulation with or without KHF treatment. **(B)** Lactate levels in the culture supernatants of RAW264.7 cells under LPS stimulation with or without KHF treatment. **(C)** Effects of GLUT1 inhibition on the expression of IL-1β, IL-6, and TNF-α in LPS-stimulated RAW264.7 cells. **(D)** Effects of GLUT1 inhibition on the expression of SLC2A1, HIF-1α, and HK2 in LPS-stimulated RAW264.7 cells. Data are presented as mean ± SD. n=3 per group. **P < 0.01 vs. model group; ^#^*P* < 0.05, ^##^*P* < 0.01 vs. KHF group. NS, *P* > 0.05.

Based on the above findings, we hypothesized that KHF suppresses macrophage activation by inhibiting GLUT1-mediated glycolysis. To test this hypothesis, RAW264.7 macrophages were stimulated with LPS in the presence or absence of KHF. LPS stimulation markedly induced the mRNA expression of SLC2A1, HIF-1α, and the downstream glycolytic enzyme HK2, indicating activation of glycolytic metabolism. KHF treatment suppressed the expression of these genes in a dose-dependent manner (**Figure 5A**). Lactate production in the culture supernatants was further measured to evaluate glycolytic activity. LPS stimulation promoted lactate accumulation, whereas KHF treatment reduced lactate levels in a dose-dependent manner, indicating inhibition of glycolytic flux (**Figure 5B**), To assess the involvement of GLUT1, the specific SLC2A1 inhibitor STF-31 was applied. Both KHF and STF-31 reduced the mRNA expression of pro-inflammatory cytokines IL-1β, IL-6, and TNF-α, as well as glycolysis-related genes SLC2A1, HIF-1α, and HK2. Co-treatment with KHF and STF-31 did not produce additional inhibitory effects (**Figures 5C, D**), suggesting that KHF exerts its anti-inflammatory and anti-glycolytic effects, at least in part, through the SLC2A1-dependent pathway. Collectively, these results indicate that KHF suppresses macrophage-mediated inflammatory responses by targeting GLUT1 and inhibiting HIF-1α–dependent glycolysis.

### 3.6 Rutin is the key active component of KHF targeting GLUT1

**Figure 6:**
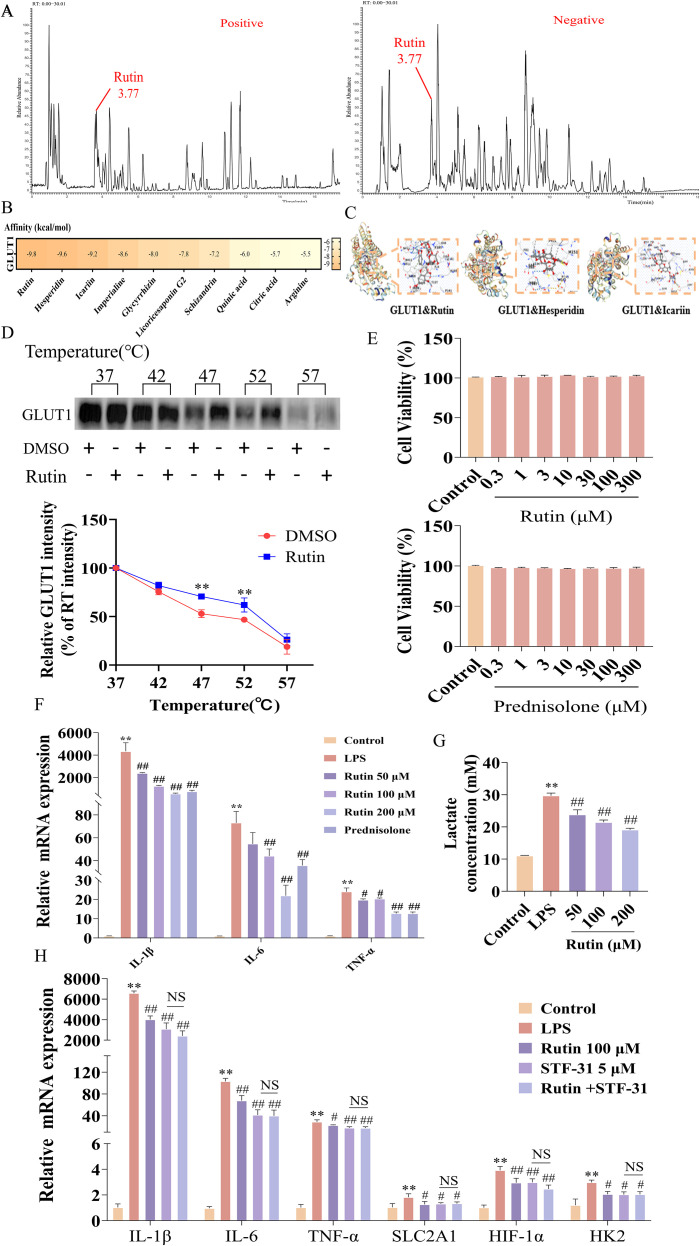
Rutin is a key bioactive constituent of KHF targeting GLUT1. **(A)** Base peak chromatograms of KHF in positive and negative ion modes obtained by UPLC–MS/MS. **(B)** Heatmap visualization of binding affinities between the top-ranked candidate compounds and GLUT1 based on molecular docking analysis. **(C)** Schematic representation of the binding conformations of the top three compounds with GLUT1. **(D)** CETSA showing the thermal stabilization of GLUT1 upon Rutin treatment. **(E)** Effects of Rutin and prednisolone on RAW264.7 cell viability. **(F)** Effects of Rutin on the relative mRNA expression of IL-1β, IL-6, and TNF-α in LPS-stimulated RAW264.7 cells. **(G)** Effects of Rutin on lactate levels in the culture supernatants of LPS-stimulated RAW264.7 cells. **(H)** Effects of GLUT1 inhibition on the expression of IL-1β, IL-6, and TNF-α and glycolysis-related genes SLC2A1, HIF-1α, and HK2 in RAW264.7 cells. Data are presented as mean ± SD. n=3 per group. *P < 0.05, **P < 0.01 vs. model group; *^#^P* < 0.05, ^##^*P* < 0.01 vs. KHF group. NS, *P* > 0.05.

To identify the key bioactive constituents of KHF responsible for its pharmacological effects, we analyzed its chemical composition using LC–MS/MS. The total ion chromatograms in both positive and negative ion modes are shown in **Figure 6A**, in which Rutin exhibited a prominent peak, suggesting its relatively high abundance in KHF. Based on relative response intensity, the top 30 compounds were selected for further analysis (**Table 2**). To further explore the interactions between these candidate compounds and GLUT1, molecular docking analysis was performed for the top 10 compounds, and the binding affinities were ranked according to binding energy and visualized using a heatmap (**Figure 6B**). Among these candidates, Rutin exhibited the lowest binding energy, indicating the strongest binding affinity to GLUT1. The binding conformations of the top three compounds are presented in **Figure 6C**. To validate the direct interaction between Rutin and GLUT1, a CETSA was performed. Rutin treatment enhanced the thermal stability of GLUT1, with significant stabilization observed at 47 °C and 52 °C, indicating a direct binding interaction (**Figure 6D**), The cytotoxicity of Rutin was evaluated using a CCK-8 assay, which showed no detectable cytotoxic effects on RAW264.7 cells at the tested concentrations (**Figure 6E**). Rutin suppressed the mRNA expression of pro-inflammatory cytokines, including IL-1β, IL-6, and TNF-α, in a concentration-dependent manner (**Figure 6F**). Lactate production was further assessed to evaluate glycolytic activity. Rutin reduced lactate accumulation in a dose-dependent manner, indicating inhibition of glycolytic flux (**Figure 6G**), To determine whether these effects are mediated through GLUT1, the specific SLC2A1 inhibitor STF-31 was applied. Both Rutin and STF-31 reduced the expression of pro-inflammatory cytokines (IL-1β, IL-6, and TNF-α) and glycolysis-related genes (SLC2A1, HIF-1α, and HK2). Co-treatment with Rutin and STF-31 did not produce additional inhibitory effects (**Figure 6H**), suggesting a shared regulatory mechanism. Collectively, these findings indicate that Rutin directly targets GLUT1, thereby suppressing HIF-1α–mediated glycolysis and attenuating inflammatory responses.

**Table 2:** Top 30 relative responses of chinese medicine components.

Number	RT	Ion mode	Identification	m/z	Formula	Class
1	4.115	[M-H]-|[M+H]+|[M+Na]+	Hesperidin	609.18481|611.19739|633.18048	C28H34O15	Flavonoids
2	8.892	[M-H]-|[M+H]+|[M+Na]+	Glycyrrhizin	821.39893|823.40826|845.3869	C42H62O16	Prenol lipids
3	1.487	[M-H]-	Citric acid	191.0209	C6H8O7	Carboxylic acids and derivatives
4	1.087	[M-H]-	Quinic acid	191.05589	C7H12O6	Organooxygen compounds
5	11.45	[M-H2O+H]+|[M+Na]+|[M+H]+|[M+NH4]+	Schizandrin	415.2121|455.20413|433.21985|450.24536	C24H32O7	Tannins
6	3.77	[M-H]-|[M+H]+	Rutin	609.14716|611.15948	C27H30O16	Flavonoids
7	5.219	[M-H]-	sphenostylisin B	659.23376	C40H36O9	---
8	5.577	[M+HCOO]-|[M+H]+|[M+Na]+	Icariin	721.23669|677.24323|699.22406	C33H40O15	Flavonoids
9	11.961	[M+H]+	2-(3,4,5-Trimethoxyphenyl)-5,6,7,8-tetramethoxy-4H-1-benzopyran-4-one	433.15002	C22H24O9	Flavonoids
10	4.472	[M+H]+	Imperialine	430.33157	C27H43NO3	Steroids and steroid derivatives
11	8.062	[M-H]-	3,8-dihydroxy-1-methylanthraquinone-2-carboxylic acid	297.0415	C16H10O6	---
12	0.985	[M+H]+	Arginine	175.11989	C6H14N4O2	Carboxylic acids and derivatives
13	7.866	[M-H]-	Licoricesaponin G2	837.39307	C42H62O17	Prenol lipids
14	7.55	[M-H]-	9,10,13-TriHOME	329.23398	C18H34O5	Fatty Acyl
15	3.712	[M+H]+	Secocularidine	342.17172	C20H23NO4	Benzoxepines
16	1.013	[M-H]-	Gluconic acid	195.05197	C6H12O7	Organooxygen compounds
17	11.09	[M+H]+	Nobiletin	403.13821	C21H22O8	Flavonoids
18	3.651	[M]+	Magnocurarine	314.17487	C19H24NO3+	Isoquinolines and derivatives
19	1.192	[M-H]-|[M+NH4]+	Turanose	341.10696|360.15073	C12H22O11	Fatty Acyl
20	1.538	[M+H]+	D-Cathinone	150.09195	C9H11NO	Organooxygen compounds
21	1.261	[M+H]+	D-proline betaine	144.10286	C7H13NO2	Carboxylic acids and derivatives
22	1.123	[M+H]+|[M-H]-	Proline	116.07198|114.05462	C5H9NO2	Carboxylic acids and derivatives
23	13.456	[M-H]-	Apigenin	269.04568	C15H10O5	Flavonoids
24	5.522	[M-H]-	Emodin 8-glucoside	431.09793	C21H20O10	Anthracenes
25	9.803	[M+H]+	Icariside I	531.18835	C27H30O11	Flavonoids
26	9.253	[M+H]+	Stellettin J	453.33707	C30H44O3	Prenol lipids
27	3.963	[M-H]-	Narirutin	579.17139	C27H32O14	Flavonoids
28	3.922	[M-H]-	Kaempferol 3-O-rutinoside	593.15198	C27H30O15	Flavonoids
29	9.762	[M-H]-	Rhein	283.02393	C15H8O6	Anthracenes
30	3.866	[M-H]-|[M-H]-	Forsythiaside	623.19623|623.19977	C29H36O15	Cinnamic acids and derivatives

## 4 Discussion

TCM formulas typically exert their therapeutic effects through the coordinated action of multiple components acting on diverse molecular targets and pathways. These interactions form a regulatory network in which multiple active constituents collectively contribute to therapeutic outcomes. Such a multi-dimensional mode of action may contribute to efficacy in complex chronic diseases while potentially limiting the adverse effects associated with single-target interventions, reflecting the TCM principle of holistic regulation and concurrent treatment of symptoms and underlying causes. In this study, using an AE-IPF mouse model, histological and biochemical analyses, including HE staining, Masson’s trichrome staining, IF, ELISA, and IHC, demonstrated that KHF markedly alleviated inflammatory responses and fibrotic alterations. Furthermore, SPC immunofluorescence analysis suggested that KHF partially restored alveolar epithelial cell status, indicating a potential protective effect on alveolar epithelial integrity. Notably, the medium dose used in this study corresponds to the clinically relevant dosage based on human equivalent dose conversion, supporting the translational relevance of the findings. Nevertheless, the complex composition and multi-target nature of TCM formulas continue to pose challenges for mechanistic elucidation and quality control.

Glycolysis plays a central role in cellular energy metabolism and biosynthetic processes. GLUT1 functions as a major glucose transporter responsible for cellular glucose uptake, thereby supporting glycolytic metabolism (23). Alterations in GLUT1 expression influence cellular glucose utilization and, consequently, glycolytic flux. Under pathological conditions, transcription factors such as HIF-1α upregulate GLUT1 expression, enhancing glucose uptake and driving metabolic reprogramming toward glycolysis (24). In addition, GLUT1 activity affects key glycolytic enzymes, including hexokinase, phosphofructokinase, and pyruvate kinase (25). Previous studies have shown that inhibition of glycolysis using 2-deoxy-D-glucose(2-DG) in LPS-induced acute lung injury models suppresses alveolar macrophage glycolytic activity, reduces lactate accumulation and pro-inflammatory cytokine production, and alleviates neutrophil infiltration and pulmonary edema (26). In AE-IPF, upregulated GLUT1 has been reported to exacerbate alveolar inflammation and collagen deposition via the lactate–absent in melanoma 2 (AIM2)/NOD-like receptor family pyrin domain containing 3 (NLRP3) inflammasome axis, whereas inhibition of GLUT1 or lactate production attenuates disease exacerbation (27). Despite accumulating evidence implicating glycolysis in AE-IPF progression, pharmacological strategies directly targeting this pathway remain limited. Current antifibrotic agents, such as nintedanib (28) and pirfenidone (29), primarily act by inhibiting fibroblast activation and extracellular matrix deposition. In contrast, the present study demonstrates that KHF exerts therapeutic effects by modulating macrophage glycolysis and inflammatory responses, representing a distinct upstream regulatory mechanism. These complementary modes of action suggest that KHF, or its active component Rutin, may provide additional therapeutic benefits by targeting metabolic and inflammatory dysregulation. Therefore, combining metabolic and anti-inflammatory interventions with conventional antifibrotic therapies may represent a promising strategy to enhance overall treatment efficacy. Such an approach could offer a more comprehensive therapeutic framework for AE-IPF by simultaneously addressing both inflammatory initiation and fibrotic progression. Building on this rationale, network pharmacology integrated with experimental validation was applied to explore the mechanisms underlying KHF activity in AE-IPF. Network pharmacology analysis identified 241 overlapping targets between KHF and AE-IPF, with enrichment in inflammation-related and metabolic pathways, including PI3K–Akt and HIF-1α signaling pathways. Given the predictive nature of network pharmacology, DARTS assays were subsequently conducted to identify direct protein targets of KHF. Mass spectrometry analysis revealed candidate proteins predominantly enriched in glycolysis-related pathways. Integrative analysis of network pharmacology and DARTS results consistently identified GLUT1 as a shared target. In addition, proteomic analysis of lung tissues further confirmed that KHF modulates glycolysis-associated protein expression *in vivo*. Together, these findings provide converging evidence that GLUT1 represents a key regulatory target mediating the therapeutic effects of KHF.

HIF-1α is a key transcription factor governing cellular adaptation to hypoxia and inflammation and is frequently aberrantly activated in inflamed and fibrotic lung tissue (30, 31). HIF-1α promotes glycolysis by transcriptionally activating downstream targets, including GLUT1 and HK2 (32). However, increasing evidence indicates that metabolism locally regulates HIF-1α activity, suggesting the presence of bidirectional crosstalk between glycolysis and hypoxia signaling (33). In the present study, using LPS-stimulated RAW264.7 macrophages, we observed that KHF significantly reduced the expression of GLUT1, HIF-1α, and HK2. This concurrent downregulation does not fully conform to a strictly linear regulatory model in which HIF-1α functions solely upstream of GLUT1. In this context, glycolytic metabolism may, in turn, influence HIF-1α stability through metabolic feedback mechanisms. For instance, lactate, a key product of glycolysis, has been reported to stabilize HIF-1α by inhibiting its degradation, whereas suppression of glycolytic flux may reduce HIF-1α accumulation (34). Based on these observations, we speculate that KHF-mediated inhibition of GLUT1 may reduce glycolytic flux, thereby secondarily affecting HIF-1α stability through metabolic feedback mechanisms. However, the precise regulatory relationship between GLUT1 and HIF-1α remains to be further clarified. Consistently, pharmacological inhibition of GLUT1 with STF-31 also suppressed the expression of HIF-1α and HK2. Notably, combined treatment with KHF and STF-31 did not produce additional inhibitory effects compared with either treatment alone, suggesting that they may, at least in part, act through a shared GLUT1-dependent pathway. Nevertheless, the lack of an additive effect does not definitively confirm direct mechanistic overlap. Collectively, these findings provide pharmacological evidence supporting the involvement of GLUT1-associated metabolic regulation in mediating the anti-inflammatory effects of KHF.

To further identify the active constituent responsible for these effects, LC-MS/MS analysis, molecular docking, and experimental validation were conducted. Among the identified components, Rutin exhibited the strongest binding affinity to GLUT1. Notably, CETSA analysis demonstrated that Rutin enhanced the thermal stability of GLUT1, providing direct evidence of target engagement. Functionally, Rutin reproduced the anti-inflammatory and glycolysis-inhibitory effects observed with KHF treatment, including suppression of pro-inflammatory cytokines and reduction of lactate production. However, It should be noted that the effective concentration of Rutin used *in vitro* cannot be directly equated to its theoretical content within the KHF formula. Under the present experimental conditions, KHF (100 μg/mL) and Rutin (100 μM) exhibited comparable inhibitory effects on IL-6 secretion in macrophages, with both achieving an inhibition rate of approximately 40%. However, the current study was primarily designed to identify and validate pharmacologically active components and their mechanisms of action, rather than to establish a quantitative equivalence between individual compounds and the whole formula. Therefore, dose–effect relationships and the precise contribution of Rutin within the complex formulation warrant further investigation. Consistent with the multi-component nature of TCM, KHF is likely to exert its pharmacological effects through synergistic interactions among multiple constituents, with different compounds contributing complementary anti-inflammatory and antifibrotic activities. Future studies integrating quantitative compositional analysis and pharmacokinetic evaluation will be necessary to further clarify the relative contribution of Rutin and the cooperative mechanisms underlying KHF efficacy. Nevertheless, as a representative bioactive flavonoid, Rutin exhibits properties that support its potential as a lead compound for the development of glycolysis-targeted therapies in AE-IPF.

Several limitations of this study should be acknowledged. First, although pharmacological evidence supports the involvement of GLUT1, genetic validation *in vivo* was not performed. Future studies using myeloid-specific GLUT1 knockout models are required to determine whether the protective effects of KHF or Rutin are directly dependent on GLUT1 signaling. Second, the bleomycin-induced model reproduces key features of pulmonary fibrosis but may not fully capture the complex pathological characteristics of AE-IPF. Additional clinical and mechanistic validation would further strengthen the translational relevance of these findings. Finally, given the complex composition of KHF, the possibility that additional active constituents contribute to its therapeutic effects through complementary regulatory pathways cannot be excluded. Future studies integrating quantitative compositional analysis and pharmacokinetic evaluation will help to elucidate the synergistic interactions among key active components and clarify the material basis underlying its pharmacological effects, thereby providing a more comprehensive understanding of the mechanisms of action of KHF.

## 5 Conclusion

**Figure 7:**
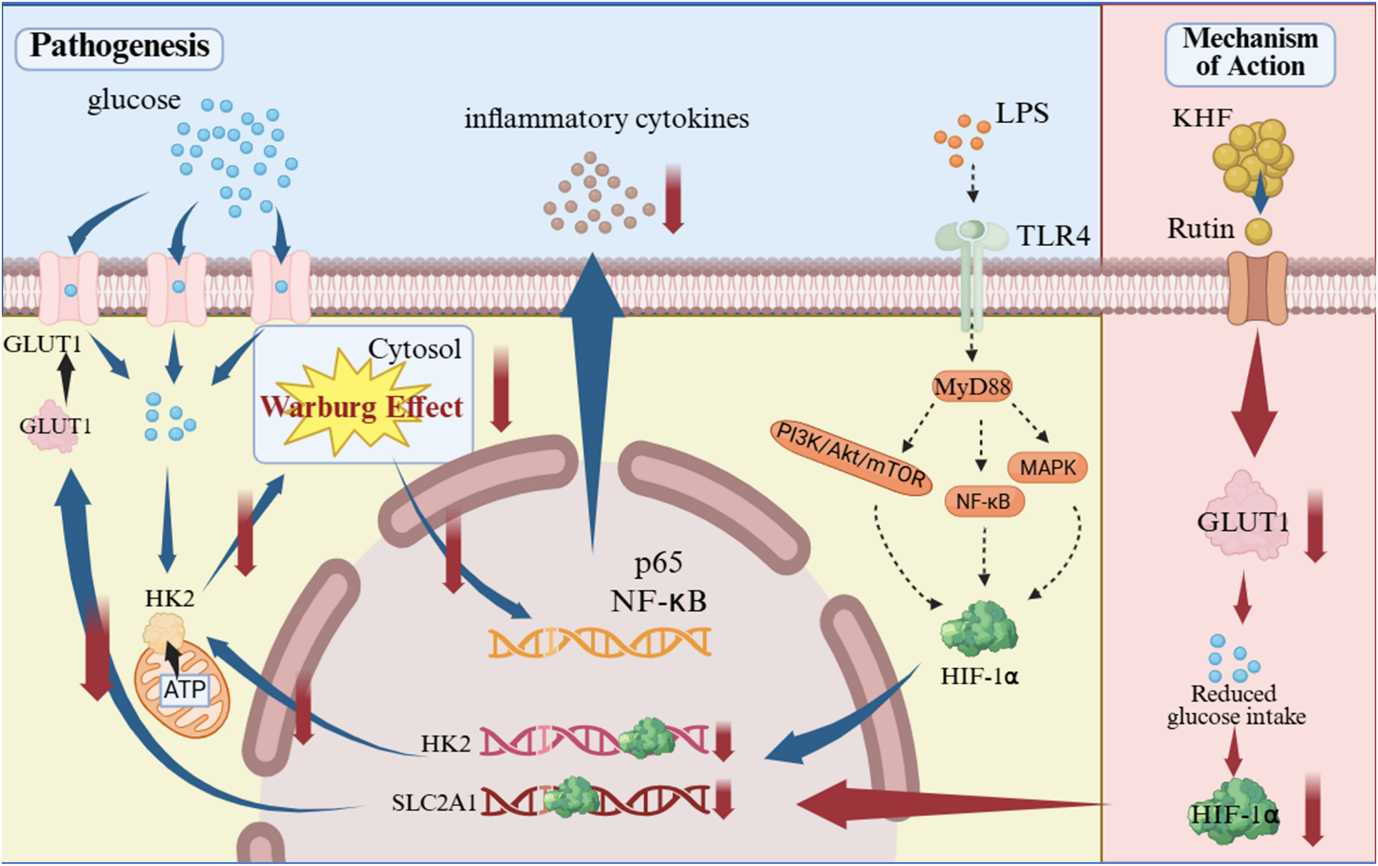
Proposed mechanism of macrophage glycolytic reprogramming in AE-IPF and the therapeutic action of KHF.

In summary (**Figure 7**), this study demonstrates that KHF exerts protective effects in AE-IPF by targeting GLUT1-associated glycolytic reprogramming and modulating the GLUT1/HIF-1α axis, thereby attenuating macrophage-driven inflammatory responses. Rutin was identified as a key bioactive constituent contributing to this metabolic regulation.

By integrating target prediction, experimental validation, and *in vivo* functional analysis, this study provides mechanistic insight into the role of glycolytic control in AE-IPF pathogenesis. These findings highlight GLUT1-centered metabolic pathways as promising therapeutic targets and support the potential clinical application of KHF in AE-IPF.

## Data Availability

The data presented in the study are deposited in the Zenodo repository, accession number 10.5281/zenodo.19609029.
